# A randomised controlled trial of preventive spinal manipulation with and without a home exercise program for patients with chronic neck pain

**DOI:** 10.1186/1471-2474-12-41

**Published:** 2011-02-08

**Authors:** Johanne Martel, Claude Dugas, Jean-Daniel Dubois, Martin Descarreaux

**Affiliations:** 1 Département de chiropratique, Université du Québec à Trois-Rivières, Trois-Rivières, G9A 5H7, Canada; 2 Département des sciences de l'activité physique, Université du Québec à Trois-Rivières, Trois-Rivières, G9A 5H7, Canada

## Abstract

**Background:**

Evidence indicates that supervised home exercises, combined or not with manual therapy, can be beneficial for patients with non-specific chronic neck pain (NCNP). The objective of the study is to investigate the efficacy of preventive spinal manipulative therapy (SMT) compared to a no treatment group in NCNP patients. Another objective is to assess the efficacy of SMT with and without a home exercise program.

**Methods:**

Ninety-eight patients underwent a short symptomatic phase of treatment before being randomly allocated to either an attention-group (n = 29), a SMT group (n = 36) or a SMT + exercise group (n = 33). The preventive phase of treatment, which lasted for 10 months, consisted of meeting with a chiropractor every two months to evaluate and discuss symptoms (attention-control group), 1 monthly SMT session (SMT group) or 1 monthly SMT session combined with a home exercise program (SMT + exercise group). The primary and secondary outcome measures were represented by scores on a 10-cm visual analog scale (VAS), active cervical ranges of motion (cROM), the neck disability index (NDI) and the Bournemouth questionnaire (BQ). Exploratory outcome measures were scored on the Fear-avoidance Behaviour Questionnaire (FABQ) and the SF-12 Questionnaire.

**Results:**

Our results show that, in the preventive phase of the trial, all 3 groups showed primary and secondary outcomes scores similar to those obtain following the non-randomised, symptomatic phase. No group difference was observed for the primary, secondary and exploratory variables. Significant improvements in FABQ scores were noted in all groups during the preventive phase of the trial. However, no significant change in health related quality of life (HRQL) was associated with the preventive phase.

**Conclusions:**

This study hypothesised that participants in the combined intervention group would have less pain and disability and better function than participants from the 2 other groups during the preventive phase of the trial. This hypothesis was not supported by the study results. Lack of a treatment specific effect is discussed in relation to the placebo and patient provider interactions in manual therapies. Further research is needed to delineate the specific and non-specific effects of treatment modalities to prevent unnecessary disability and to minimise morbidity related to NCNP. Additional investigation is also required to identify the best strategies for secondary and tertiary prevention of NCNP.

**Trial registration:**

ClinicalTrials.gov: NCT00566930

## Background

Non-specific neck pain is frequent, with an annual prevalence estimated to be 30% to 50%[1]. Often persistent or recurrent, neck pain is still being reported by 50% to 85% of patients 1 to 5 years after initial onset[2]. Its course is usually episodic, and improvement is of variable degrees between episodes, but complete recovery is unusual for most patients[3]. Manual therapy (mobilisation or manipulation), exercise intervention, low-level laser therapy and, to a lesser extent, acupuncture, are more effective than no treatment, sham, or alternative interventions to stop episodes of neck pain. None of these strategies is, however, superior to any other[4]. Evidence also indicates that supervised exercises with or without manual therapy are better than usual or no care[4] and that a multimodal care approach combining exercise with manual therapy seems to be beneficial for non-specific chronic neck pain (NCNP)[5]. Based on care episodes of 6 to 8 weeks with various blends of non-invasive interventions,[4] no particular course of care improves the prognosis or appreciably affects the natural history of neck disorder or its recurrence. Evidence for the effectiveness of neck pain prevention strategies is therefore lacking.

Chiropractic intervention is usually directed toward neuromusculoskeletal problems,[6] with around 25% of patients presenting to a chiropractor and complaining of neck pain[6,7]. Spinal manipulative therapy (SMT) is the chiropractor's main therapeutic tool. A holistic paradigm is, however, at the foundation of this profession[8,9]. A recent review of chiropractic preventive care (CPC)[10] indicates that more than 90% of surveyed chiropractors believe that it is helpful to patients. In the realm of public health, CPC is a strategy of tertiary prevention. In clinical practice, it is scheduled at regular intervals, is usually elective and is not based on the occurrence of symptoms. It is typically initiated after the resolution of a clinical problem, and is designed to preserve optimum health while minimizing recurrence. Only 1 randomised controlled trial (RCT) investigating CPC efficacy in a non-specific low back pain population has been published[11]. The results indicated that the group receiving preventive SMT maintained improvement in disability level during the symptomatic period of care while the control group returned to its pre-trial disability level. However, no differences between the two groups were observed for pain. Other literature regarding CPC relates to surveys, focus groups, reviews or editorials,[10,12-20], and many important characteristics of CPC have not been studied, such as its prevalence of use, its clinical indication, its effectiveness and its acceptance by patients[10].

The objective of the study is to investigate the efficacy of preventive SMT compared to a no treatment group in NCNP patients. Another objective is to assess the efficacy of SMT with and without a home exercise program. It was hypothesised that during a 10-month period of preventive care, participants with NCNP receiving SMT combined with a home exercise program would have less pain and disability with improved function compared to patients given only SMT or no treatment.

## Methods

### Participants

Study participants were recruited if they had NCNP, defined as pain of mechanical origin located in the anatomical region of the neck, with or without radiation to the head, trunk or limbs, as described by Guzman et al[3]. The inclusion criteria were: aged between 18 and 60 years, neck pain lasting 12 weeks or more, no physical therapy, not currently under chiropractic care or rehabilitation for the neck area, willingness to adhere to the treatment protocol, and signed informed consent. Participants with concurrent headaches, non-radicular pain in the upper extremities and lower back pain were not excluded if neck pain was the main symptom. The exclusion criteria included neck pain due to a motor vehicle accident, neck surgery, severe osteoarthritis and inflammatory arthritis, neurological, cardiovascular, infectious metabolic and endocrine diseases, pregnancy and any cardinal signs of potential vertebral artery dissection.

Participants were recruited through radio and printed advertisements aimed at the French-speaking community of Trois-Rivières, Québec (population 126,000). The study was carried out entirely at the chiropractic clinic and human research laboratory of the Department of Chiropractic at the Université du Québec à Trois-Rivières. All participants gave their informed written consent according to the research protocol approved by the local ethics committee.

Three research assistants undertook standardised phone screening regarding major inclusion and exclusion criteria for study participation. Three other research assistants, who were experienced chiropractors and were blinded to future treatment allocation, performed the preliminary standardised history-taking and physical examination of eligible participants. Radiographs of the cervical spine were ordered when indicated[21]. All preliminary outcome measures were collected at this point.

### Randomisation and blinding

The trial was divided into 2 phases. The first was the non-randomised, symptomatic phase during which all eligible participants received a short course of SMT. After completing the symptomatic phase, participants were randomly assigned to one of three parallel groups. The second phase, the preventive phase, lasted 10 months.

Participants had an equal probability of assignment to any 1 of the 3 groups. Each study participant was assigned a number by the principal investigator (PI) who put these numbers on individual cards in an opaque envelope. An assistant, not involved in the research project and blinded to the process, drew numbers from the original, opaque envelope and arranged them in 3 different opaque envelopes which were numbered 1 to 3. Randomisation was concealed until the beginning of the preventive treatment phase. No block or stratification strategy was used in the randomisation process.

Randomisation was carried out after acceptance of the appropriate number of participants into the study and before beginning the non-randomised, symptomatic phase. The PI opened the sealed, opaque envelopes after the symptomatic phase was over, and baseline (second set) outcome measures were collected. Each participant was assigned to his/her appropriate group before beginning the preventive phase of the trial. They remained on the same allocation throughout the entire period if they continued in the trial.

A credible placebo for SMT of patients previously administered this type of therapy does not exist, especially if its specific and non-specific effects are considered[22-24]. Therefore, neither the participants, the treating chiropractors nor the assessors were blinded to treatment allocation for the future preventive phase of the trial. Only the data analyst was blinded to treatment allocation.

### Interventions

During the symptomatic phase of the trial, all eligible participants received a short course of SMT designed to relieve symptoms. A team of 3 chiropractors using high velocity low amplitude spinal manipulation[8] and with at least 3 years of experience was responsible for treating the study participants. The interventions were standardised beforehand and lasted 10 to 15 minutes. Between 10 and 15 treatments were provided over a 5- to 6-week period. Each treatment consisted of a maximum of 4 spinal manipulations to the cervical and upper thoracic areas (down to T4). Myofascial soft tissue therapy (brief trigger point therapy) was permitted but was to be kept to a minimum. No advice or educative strategies were allowed. At this stage, the chiropractors and study participants were blinded to treatment allocation for the preventive phase.

The preventive phase of the trial lasted 10 months during which the participants attended the clinic regularly. A chiropractor with at least 3 years of experience was in charge of the interventions for each group. The interventions were standardised beforehand. Medication(s) and co-intervention such as other manual therapies, physiotherapy, massage therapy as well as any other common neck pain treatment were discouraged. Before adopting any such pain control strategy, the participants were instructed to first call their treating chiropractor to discuss the problem. Patients in all 3 groups received identical verbal and written instructions regarding co-interventions and were asked to record any co-interventions in a personal diary. Patients were given an ice pack that could be used whenever pain intensity reached a level of 5/10. Again all 3 groups received identical instructions concerning ice application.

At each visit, the chiropractor asked the participants for a standardised, short health history regarding symptoms during the last period and had them complete a visual analog scale (VAS) for current symptoms. The chiropractor also performed standardised passive palpation of the cervical and upper thoracic spine. No advice or educative strategy was allowed during this phase, nor was any type of soft tissue therapy. Diaries were distributed at these visits. Other interventions during each visit were specific for each of the 3 groups:

### Spinal manipulation group

This group received a maximum of 4 spinal manipulations to the cervical and upper thoracic areas. They were given 1 treatment per month that lasted 10 to 15 minutes.

### Spinal manipulation combined with a home exercise program group

This group received a maximum of 4 spinal manipulations to the cervical and upper thoracic areas (down to T4). They were dispensed with 1 treatment per month, and each of them lasted 10 to 15 minutes.

Participants were advised to perform a home exercise program at least 3 times a week. The program was designed by an experienced kinesiologist and required low technology equipment (elastic tubing and foam physioballs). It included general range of motion (ROM) exercises that served for warm-up and cool down purposes, followed by 4 stretching/mobilization and 4 strengthening exercises (concentric and isometric contractions) of the cervical and upper thoracic spine, principally flexion/extension, lateral flexion and rotation of the cervical spine. Three series of each exercise were performed during a training session, with a 30- to 60-second rest period between each series. A complete training session lasted between 20 to 30 minutes.

All participants were instructed to follow the same exercise routine. However, exercise volume was tailored to each participant's strength and flexibility as well as his/her ability to complete the routine with minimal neck pain. Each patient received a written copy of the program. At trial onset, a kinesiologist met each participant individually on 2 different occasions to instruct and correct them on execution of the exercises. Between these 2 meetings, follow-up was conducted through phone interviews to answer questions and confirm that the exercises were not triggering cervical pain. The kinesiologist met the participants individually every 2 months (during the regular treatment visit) to ensure full understanding, appropriate execution and compliance. Minor changes were made only when exercises produced symptoms or when a patient was unable to perform a specific exercise. During the 10 months of the preventive phase, exercise type and volume could be modified under the following conditions: exercise-related pain described by the patient or major difficulty in carrying out an exercise.

### Attention-control group

This group received no treatment (no SMT or exercise program) but each participant attended the clinic once every 2 months. To give all trial participants the same attention, each visit lasted twice as long as the other two groups treatment time, specifically 20 to 30 minutes. The same procedures as for the other 2 groups were performed at these meetings (standardised short health history, VAS, standardised passive palpation and the distribution of diaries) but with much slower flow. As with the 2 other groups, no advice or educative strategy was allowed.

### Outcomes

#### Primary and secondary outcome measures

The primary outcome measure throughout the trial was pain level. Pain was scored with a 10-cm VAS. The psychometric properties of the VAS have been studied extensively[25]. The number of patients that stayed below a level of clinically acceptable pain (2 point difference from the symptomatic phase baseline VAS score) during the preventive phase of the trial was also assessed. For example, a patient that went from a score of 5 to 3 on the VAS during the symptomatic phase and maintained or improved this score throughout the preventive phase was considered to show a clinically meaningful response. Function and disability were considered the secondary outcomes in the study. Cervical spine function was assessed with the cervical range of motion instrument (cROM^©^). Many studies have demonstrated acceptable validity and reliability of this instrument[26]. Active cervical bilateral rotation, bilateral lateral flexion, and flexion and extension were measured with the patient in the seated position. The patient performed each active cROM three consecutive times in sequence-specific order. The mean of the 3 consecutive readings was used in this study. Disability was measured with the Neck Pain Disability Index (NDI) and the Bournemouth Questionnaire (BQ). The psychometric properties of these 2 instruments, including their translation into the French language[27,28] have been thoroughly assessed and are adequate for such a trial[28-32].

#### Exploratory outcome measures

Exploratory outcome measures included health-related quality of life (HRQOL), fear and avoidance phenomena, exercise adherence and co-intervention. HRQOL and fear avoidance phenomena were scored with the SF-12 Questionnaire[33] and the Fear-avoidance Behaviour Questionnaire (FABQ),[34] respectively, whose psychometric properties are adequate for such a trial. The physical health composite score and the mental health score from the SF-12 were calculated.

Exercise adherence and co-intervention were measured through a diary that patients completed on a weekly basis. Use of co-intervention, registered by the patients, was calculated by adding up the number of co-intervention episodes from one visit to another. Also recorded were the frequency of ice application due to pain intensity greater than 5/10 and the use of analgesics or consultation with other professionals for pain management. Patients performing home exercises also recorded the dates of their 3 weekly exercise sessions.

#### Outcome measures

Preliminary data were collected after participants signed an informed consent form, before the beginning of the non-randomised, symptomatic phase of the trial. Baseline data were gathered once the symptomatic phase was over, on specific appointment. During the 10 months of the preventive phase, all outcomes were noted every 2 months. For the groups receiving treatments, these measurements were recorded before the treatments.

For preliminary and baseline data, the assessors were blinded to group allocation. The assessors were not blinded to the data during the preventive phase but were trained before the trial to ensure standardisation of the outcome measures.

### Statistical analysis

#### Sample size

Thirty-five participants per group were required to have a 90% chance of detecting a significant difference between groups in the mean of VAS scores (i.e. a 2-point difference on the VAS at the 2-sided 5% level) with an assumed standard deviation of 2.5 and a loss to follow-up of 20%. Since no RCT on the efficacy of preventive spinal manipulation for chronic cervical pain has been performed in the past, the 20% loss to follow-up level was estimated from the results of a similar RCT on the efficacy of preventive spinal manipulation for chronic lumbar pain[11]. The effect size was also estimated from this trial.

### Statistical methods

All data were analysed according to a pre-established experimental design using Version 6.1 of Statistica software. One-way ANOVA was performed for baseline values of continuous variables. T-tests for dependent samples were conducted on primary and secondary outcomes to analyse data from the symptomatic phase of the trial (pooled data).

The main analysis was undertaken on an intention-to-treat basis. Missing values were imputed on the basis of the last-observation-carried-forward technique and included all randomly-assigned participants who stayed in the study until the first visit of the preventive phase. All clinical variables were analyzed using ANCOVA, with treatment and time intervals representing the main factors. Gender, age and pain improvement in the symptomatic phase were used as covariates in the analyses. Data were adjusted for gender and pain improvement in the symptomatic phase because these 2 variables were deemed to play an important role in possible further improvement of the patient's condition. One-way ANOVA served to compare co-intervention data across the 3 groups.

Whenever factorial analyses revealed significant effects, post hoc analyses were performed with the least significant difference test. For all analyses, statistical significance was set at p < 0.05.

## Results

### Participant flow and follow-up

**Figure 1:**
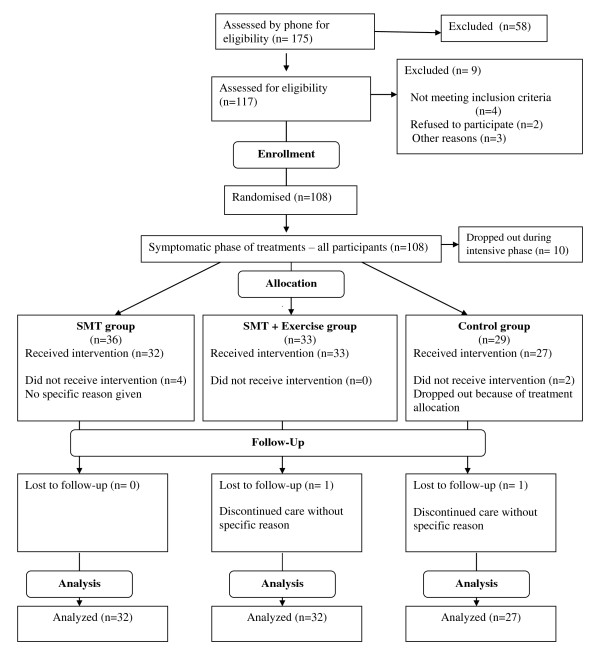
**Progression of participants through the trial including withdrawal and exclusion**.

Patients were recruited during the months of August and September 2007. One hundred and seventeen participants were assessed for eligibility through an initial evaluation in September 2007. Preliminary data were collected at this time. All included participants (n = 108) received treatments during the symptomatic phase of the trial, which began in September 2007. Ten patients (randomized but no allocated) dropped out of the study. This phase ended in December 2007, at which point baseline data were collected. After completing symptomatic treatments, participants were assigned to their groups. Drop outs from the symptomatic phase led to unequal group sizes: SMT (n = 36), SMT + exercise (n = 33) or attention-control (n = 29). The preventive phase lasted from January 2008 to October 2008, at which point all participant follow-up ended (Figure 1). There was no deviation from the protocol or any serious adverse events during the RCT.

### Baseline demographics and clinical characteristics of each group

The baseline demographics and clinical characteristics of participants (measured before the symptomatic phase of the trial), presented in Table 1, show that the percentage of males was considerably lower in the attention-control group than in the 2 experimental groups. Regarding the duration of symptoms, fewer participants from the attention-control group had pain for less than 1 year compared to the 2 experimental groups (3.7% versus 9.3% and 9.1%). The other baseline characteristics were similar between the 3 groups, indicating that randomisation was successful for these variables. The mean pain score of 3.6 cm on the VAS and the mean disability score of 23.1 on the NDI indicated the severity of complaints and the underlying disability. Active ROMs were reported for each plane and were not divided into right/left or flexion/extension.

**Table 1:** Baseline characteristics of patients enrolled in the trial

Characteristics	Spinal manipulation	Spinal manipulation + Home exercise program	Attention-control
Mean age (SD), *years*	36.8 (10.5)	43.3 (10.5)	43.3 (10.9)

Sex = male (%)	39.4%	42.4%	20.7%

Height (SD), *m*	153.8 (29.9)	155.7 (34.8)	144.9 (33.2)

Weight (SD), *kg*	65.8 (3.0)	66.1 (3.7)	64.8 (4.1)

**Category of pain duration, n (%)**			

1 - Less than 6 months	1 (3.1%)	0	0

2 - Between 6 months & 1 year	2 (6.2%)	3 (9.1%)	1 (3.7%)

3 - Between 1 & 2 years	6 (18.8%)	0	7 (25.9%)

4 - Between 2 & 3 years	7 (21.9%)	7 (21.2%)	4 (14.8%)

5 - Between 3 & 5 years	4 (12.5%)	6 (18.2%)	5 (18.5%)

6 - Between 5 & 10 years	5 (15.6%)	4 (12.1%)	8 (29.6%)

7 - More than 10 years	7 (21.9%)	13 (39.4%)	2 (7.4%)

Mean pain = VAS (SD), *cm*	3.1 (2.1)	3.8 (2.6)	3.8 (2.5)

Mean disability = NDI (SD)	21.4 (8.8)	22.2 (9.0)	25.6 (10.0)

Mean disability = BQ (SD)	26.4 (10.4)	22.4 (11.7)	27.7 (12.2)

Rotation (SD), °	117.6 (15.3)	113.0 (21.3)	114.4 (16.2)

Lateral flexion (SD), °	64.8 (17.9)	59.3 (14.8)	63.4 (13.4)

Flexion-extension (SD), °	109.7 (19.9)	104.0 (21.4)	100.8 (19.8)

SF-12, physical scale	48.7 (5.6)	50.0 (7.2)	47.3 (7.6)

SF-12, mental scale	45.3 (9.9)	44.8 (9.0)	46.7 (10.4)

FABQ-1, work-related	10.5 (8.7)	10.7 (8.0)	14.1 (7.7)

FABQ-2, physical-activity related	6.6 (5.7)	7.5 (6.2)	7.6 (6.4)

SD = standard deviation, VAS = Visual analog scale, NDI = Neck disability index, BQ = Bournemouth questionnaire, FABQ-1 = Fear-avoidance questionnaire-1 work-related, FABQ-2 = Fear-avoidance questionnaire-2 physical activity-related

### Non-randomised symptomatic phase

During the non-randomised, symptomatic phase of this trial, the average pain level of participants decreased by 1.2 cm on the VAS, the disability level declined by 4.9 points on the NDI and by 6.5 points on the BQ. Function, measured as active cROMs, improved by 6.5° in the axial plane (rotation), 2.4° in the coronal plane (lateral flexion) but decreased by 14.1° in the sagittal plane (flexion-extension). These differences were all statistically significant, except for lateral flexion (Table 2). Clinical significance thresholds have been proposed for many neck pain clinical outcomes (1/10 for VAS, 8/100 for NDI and 4.4/70 for BQ)[27,35,36]. Thus, VAS and BQ reached clinically significant changes. Clinical significance values for ROM have not been established in NCNP patients.

**Table 2:** Primary and secondary outcomes results of the symptomatic phase of treatments (non-randomised intervention).

Outcome measures	Before treatments	After treatments	Difference	p value
Mean pain: VAS (SD), *cm*	3.4 ± 2.3 [0-8.1]	2.3 ± 1.9 [0-8.5]	1.1	p = 0.0003

NDI (SD)	22.9 ± 9.3 [0-52]	18.0 ± 9.6 [0-44]	4.9	p = 0.0005

BQ (SD)	25.3 ± 11.6 [2-57]	18.9 ± 10.9 [2-56]	6.5	p = 0.0001

Range of motion (°)				

Rotation (SD)	115.2 ± 17.6 [65-151]	121.7 ± 17.4 [73-160]	6.5	p = 0.0161

Lateral flexion (SD)	63.9 ± 17.0 [28-104]	66.3 ± 15.3 [23-110]	2.4	p = 0.3423

Flexion-extension (SD)	119.4 ± 19.0 [52-166]	105.3 ± 20.5 [52-151]	14.1	p < 0.0001

Mean values, standard deviation as well as minimal and maximal values are indicated.

### Preventive phase - Primary and secondary outcomes

**Figure 2:**
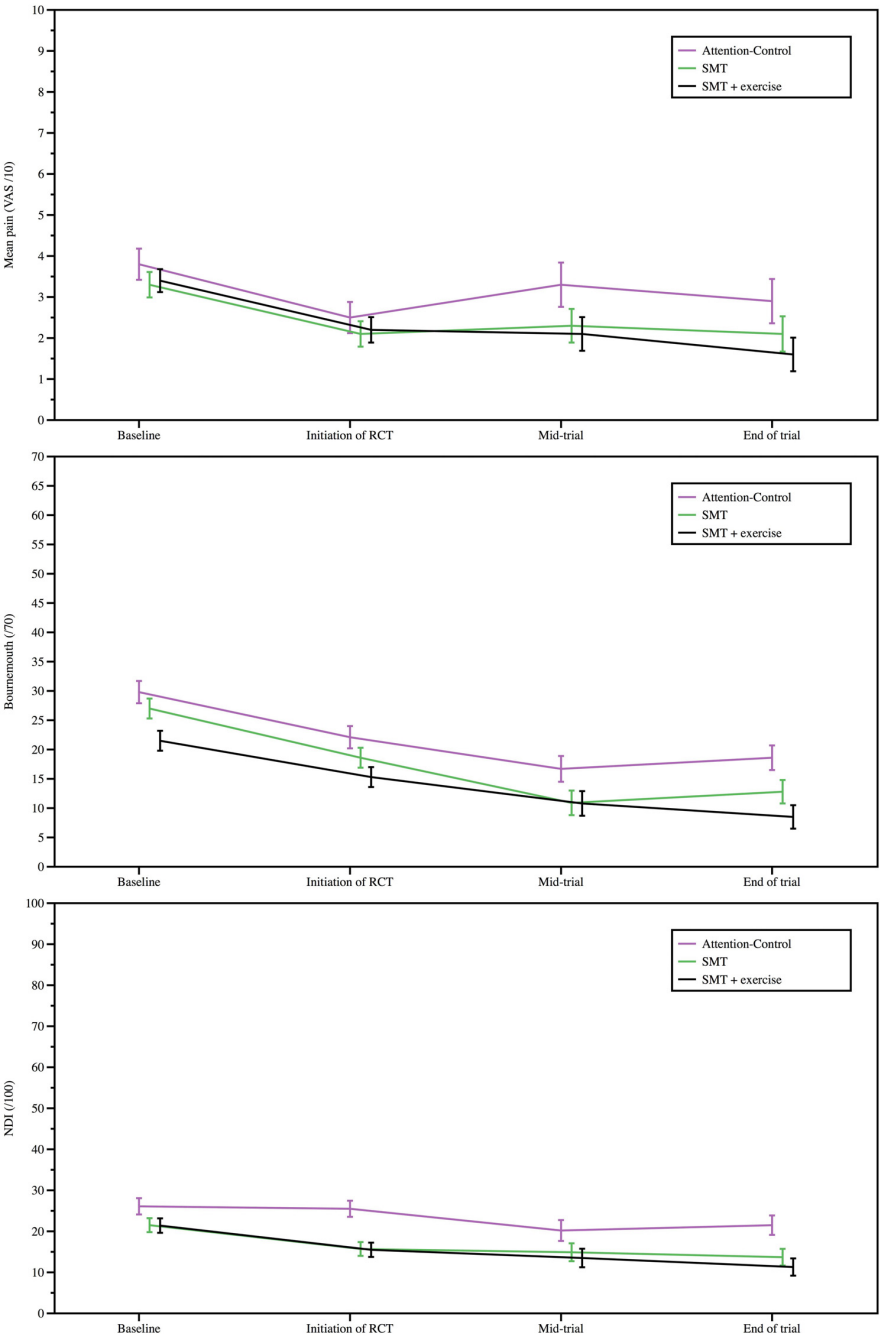
**Mean (SD) VAS, NDI and Bournemouth scores during the preventive phase of the trial**.

Although improvement was significant on the NDI, BQ and lateral flexion ROM in all groups, ANCOVA (adjusted for gender and pain improvement in the symptomatic phase) did not yield significant between group differences for the primary and secondary outcome measures. Table 3 reports mean (SD) values and 95% confidence intervals for the primary and secondary outcomes, namely, VAS, NDI, BQ and active cROMs, in the baseline, initiation of RCT, mid-trial and end of trial. Improvement in pain and disability status (NDI and BQ scores) over time is illustrated in Figure 2. Even though no significant group differences could be found, a trend was observed toward a progressive decrease in disability level for both variables. Overall a majority of the participants in the attention-control group (16 patients; 55%), the SMT group (20 patients; 56%) and the SMT + exercise group (24 patients; 73%) stayed below a level of clinically acceptable pain (2 point difference from the symptomatic phase baseline VAS score) during the preventive phase of the trial during the preventive phase of the trial.

**Table 3:** Mean (SD) values and 95% confidence interval for the primary and secondary outcomes.

Outcome measure	Group	Baseline	Initiation of RCT	Mid-trial	End of trial
Mean pain: VAS (SD), *cm*	Attention-control	3.8 ± 1.9 (3.0 to 4.5)	2.5 ± 2.1 (1.8 to 3.3)	3.3 ± 2.6 (2.2 to 4.3)	2.9 ± 2.9 (1.9 to 4.0)
	SMT	3.3 ± 1.7 (2.7 to 3.9)	2.1 ± 1.7 (1.5 to 2.7)	2.3 ± 2.3 (1.5 to 3.1)	2.1 ± 2.3 (1.2 to 2.9)
	SMT + exercises	3.4 ± 1.7 (2.9 to 4.0)	2.2 ± 1.7 (1.6 to 2.8)	2.1 ± 2.3 (1.3 to 2.9)	1.6 ± 2.3 (0.8 to 2.4)

NDI	Attention-control	26.1 ± 12.7 (21.4 to 30.9)	25.5 ± 4.5 (17.8 to 27.2)	20.2 ± 13.8 (15.0 to 25.4)	21.5 ± 14.0 (16.1 to 26.8)
	SMT	21.5 ± 10.7 (17.8 to 25.2)	15.7 ± 10.7 (12.0 to 19.4)	14.9 ± 11.8 (10.8 to 19.0)	13.7 ± 12.1 (9.5 to 17.9)
	SMT + exercises	21.4 ± 10.4 (17.8 to 25.0)	15.5 ± 10.4 (11.9 to 19.1)	13.5 ± 11.6 (9.5 to 17.5)	11.3 ± 11.8 (7.3 to 15.4)

Bournemouth	Attention-control	29.8 ± 15.4 (23.9 to 35.6)	22.1 ± 14.8 (16.6 to 27.7)	16.7 ± 11.7 (12.3 to 21.1)	18.6 ± 12.7 (13.7 to 23.4)
	SMT	27.0 ± 12.7 (22.7 to 31.4)	18.6 ± 12.1 (14.4 to 22.8)	10.9 ± 9.5 (7.6 to 14.2)	12.8 ± 10.4 (9.2 to 16.4)
	SMT + exercises	21.5 ± 13.0 (16.9 to 26.0)	15.3 ± 12.7 (10.9 to19.7)	10.8 ± 10.1 (7.4 to 14.3)	8.5 ± 11.0 (4.6 to 12.3)

Flexion-extension	Attention-control	110.0 ± 22.8 (101.5 to 118.6)	100.8 ± 21.2 (92.8 to 108.8)	106.3 ± 19.1 (99.0 to 113.5)	106.1 ± 23.3 (97.4 to 114.9)
	SMT	124.5 ± 16.5 (118.8 to 130.2)	108.5 ± 20.5 (101.5 to 115.6)	114.4 ± 22.5 (106.5 to 122.2)	114.1 ± 21.0 (107.0 to 121.3)
	SMT + exercises	124.5 ± 16.5 (118.8 to 130.2)	105.8 ± 25.1 (97.1 to 114.5)	115.5 ± 16.2 (109.9 to 121.1)	115.6 ± 22.5 (107.8 to 123.4)

Rotation	Attention-control	114.0 ± 19.1 (106.8 to 121.2)	118.1 ± 17.5 (112.1 to 124.7)	118.4 ± 15.9 (112.4 to 124.4)	119.5 ± 15.4 (113.6 to 125.3)
	SMT	117.1 ± 17.0 (111.2 - 123.0)	124.9 ± 17.6 (118.7 - 131.0)	123.4 ± 28.0 (113.7 - 133.1)	126.9 ± 29.5 (116.7 - 137.1)
	SMT + exercises	114.7 ± 24.8 (106.1 - 123.3)	120.9 ± 20.5 (113.9 - 128.0)	122.8 ± 30.3 (112.3 - 133.3)	126.7 ± 25.7 (117.8 - 135.6)

Lateral Flexion	Attention-control	63.7 ± 15.4 (58.0 - 69.5)	68.8 ± 13.2 (63.7 - 73.8)	74.7 ± 15.9 (68.8 - 80.7)	70.5 ± 11.1 (66.2 - 74.7)
	SMT	66.3 ± 20.8 (59.1 - 73.5)	68.9 ± 15.3 (63.6 - 74.2)	75.8 ± 24.5 (67.2 - 84.3)	67.1 ± 13.6 (62.5 - 71.8)
	SMT + exercises	59.4 ± 17.9 (53.2 - 65.6)	59.6 ± 17.3 (53.6 - 65.6)	68.1 ± 18.5 (61.7 - 74.5)	70.8 ± 23.7 (62.6 - 79.0)

### Preventive phase - exploratory outcomes

Significant improvements in FABQ scores (work and physical activity-related scores) over time were noted in all groups. Statistical analysis of HRQOL (physical health composite score and mental health score from the SF-12) did not yield any significant effect. Table 4 presents the mean (SD) values and 95% confidence intervals for the secondary outcomes.

**Table 4:** Mean (SD) values and 95% confidence interval for the exploratory outcomes.

Outcome measure	Group	Baseline	Initiation of RCT	Mid-trial	End of trial
FABQ 1/42*	Attention-control	14.7 ± 8.5 (11.5 to 17.9)	14.0 ± 9.0 (10.5 to 17.4)	7.8 ± 8.5 (4.4 to 11.1)	8.8 ± 9.5(5.2 to 12.4)
	SMT	10.4 ± 9.2 (7.2 to 13.6)	10.5 ± 8.1 (7.7 to 13.3)	6.9 ± 7.8 (4.2 to 9.6)	6.2 ± 8.1 (3.5 to 9.0)
	SMT + Exercises	11 ± 11.0 (7.2 to 14.8)	7.6 ± 8.7 (4.5 to 10.6)	6.1 ± 9.0 (3.2 to 9.2)	4.7 ± 9.5 (1.5 to 8.0)

FABQ 2/24**	Attention-control	8.2 ± 7.4 (5.5 to 11.0)	7.7 ± 7.4 (4.9 to 10.5)	7.2 ± 6.9 (4.6 to 9.8)	5.4 ± 5.8 (3.2 to 7.6)
	SMT	7.2 ± 6.1 (5.1 to 9.3)	6.2 ± 6.6 (3.9 to 8.5)	4.8 ± 6.1 (2.7 to 6.9)	5.7 ± 5.2 (3.9 to 7.5)
	SMT + Exercises	7.7 ± 7.8 (5.0 to 10.4)	5.8 ± 7.2 (3.2 to 8.3)	3.3 ± 6.6 (1.0 to 5.6)	2.0 ± 5.8 (0.1 to 4.0)

SF-12 (physical)	Attention-control	47.3 ± 8.5 (44.2 to 50.5)	47.5 ± 9.0 (44.1 to 50.9)	50.5 ± 9.3 (47.1 to 54.0)	52.1 ± 8.2 (49.0 to 55.2)
	SMT	48.9 ± 6.4 (46.7 to 51.1)	52.1 ± 8.4 (49.8 to 55.0)	51.1 ± 7.8 (48.5 to 53.8)	53.1 ± 6.9 (50.7 to 55.5)
	SMT + Exercises	49.9 ± 8.7 (47.0 to 52.9)	51.7 ± 7.8 (49.1 to 54.4)	52.1 ± 7.8 (49.5 to 54.8)	54.1 ± 7.2 (51.7 to 56.6)

SF-12 (mental)	Attention-control	51.3 ± 9.3 (47.7 to 54.8)	51.0 ± 11.9 (46.4 to 55.5)	52.6 ± 11.4 (48.2 to 56.9)	49.9 ± 10.1 (46.1 to 53.7)
	SMT	47.9 ± 9.8 (44.5 to 51.3)	48.3 ± 10.1 (44.8 to 51.8)	49.6 ± 9.8(46.3 to 53.0)	52.3 ± 8.4 (49.3 to 55.2)
	SMT + Exercises	47.5 ± 10.1 (43.9 to 51.0)	47.2 ± 10.1 (43.6 to 50.7)	48.5 ± 9.8(45.1 to 51.9)	49.8 ± 8.7 (46.9 to 52.8)

* FABQ 1 = Fear avoidance behaviour questionnaire - Work related score, maximum score of 42.

** FABQ 2 = Fear avoidance behaviour questionnaire - Physical activity related score, maximum score of 24.

### Compliance, exercise adherence and co-intervention

The overall compliance of each participant for all groups (excluding the patients who dropped-out during the randomized phase of the trial) was 96% (8.2) for the attention-control group and 96% (6.3) and 93% (9.8) for SMT and SMT+ exercise groups respectively. Patients' diaries were consulted to verify exercise adherence in the combined intervention group and co-intervention along with ice application in all patients. Mean adherence to the home exercise program (exercise sessions completed/total number of possible sessions) was 48.8%. Out of 26 patients in the combined intervention group, 16 (61.5%) completed more than 50% of the exercise sessions, 6 (23.1%) finished less than 50%, and 4 (15.4%) did not adhere at all to the program.

**Figure 3:**
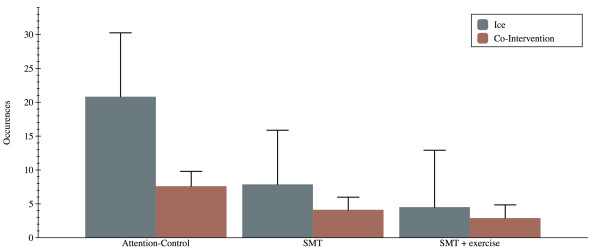
**Co-intervention and use of ice during the preventive phase of the trial**. * Indicates a significant group difference in use of ice and ** a significant group difference in co-intervention.

Significant between group differences were noted for both the number of co-intervention episodes (p = 0.006) and ice application (p = 0.032). The attention-control group required significantly more co-intervention (7.6 ± 1.1) compared to the other 2 groups (4.1 ± 0.9 and 2.9 ± 0.9). Moreover, the attention-control group chose to apply ice as a pain-relieving modality significantly more often (20.8 ± 4.7) that the other 2 groups (7.8 ± 4.0 and 4.5 ± 4.2). Figure 3 reports the co-intervention and ice-use data for all groups.

## Discussion

### Principal findings, possible mechanisms and explanation of results

This study hypothesised that participants in the combined intervention group would have less pain and disability and better function than participants from the 2 other groups during the preventive phase of the trial. In fact, all 3 groups showed primary and secondary outcomes scores similar to those obtained following the non-randomised, symptomatic phase. No significant change in HRQOL was associated with the preventive phase, but the 3 groups demonstrated statistically significant improvement in their fear avoidance behaviour scores over time. Overall spinal manipulation or spinal manipulation combined with exercises did not yield significant advantages when compared to the no treatment strategy.

The course of neck pain is usually described as episodes occurring over a lifetime with variable degrees of recovery between episodes[2,3,37]. Furthermore, prior pain episodes are associated with poorer prognosis. In our study, more than 75% of experienced NCNP, defined as recurring episodes or continuous pain, for more than 2 years at study onset. Given the course and natural history of neck pain, we expected that pain, function and disability of participants in the attention-control group would regress to pre-treatment levels during the 10 months of the preventive phase. Stabilisation of improvement in the attention-control group after the symptomatic phase raises questions regarding the causality of this change in the course of the disorder. One hypothesis relates to the attention-control group requiring more co-intervention of any type and choosing ice as a pain-relieving modality significantly more often than the 2 other groups. These additional strategies of self-management in terms of symptom attention-control and coping might explain, at least partially, the equivalence of the 3 groups in terms of pain, function and disability during the preventive phase of the trial.

Another plausible explanation for these results is the placebo effect, which refers to the outcome attributable to a procedure but not to its specific properties. Any perceived therapeutic action includes its specific and non-specific effects. The non-specific effects, also termed the "context of treatment", represent the psychosocial aspect of every treatment. In our study, different clinicians were responsible for each of the 3 groups, and therefore the treating clinician (not only the treatment used) may have influenced the clinical outcomes. Many factors contribute to these outcomes, the 3 most often described being the patient, the provider and patient-provider interaction[38-41]. From the patient's perspective, the magnitude of the placebo response is highly variable between individuals,[38,40] and patients' expectations influence treatment outcomes, including the specific and non-specific effects[41]. Chronic conditions with fluctuating courses, such as chronic neck pain, are usually more placebo-prone[39,41]. Finally, many studies indicate that patient-provider interaction is a potent factor in health outcomes[38,39,41]. Many factors, such as a clear diagnosis, an opportunity for dialogue or the overall "context of treatment", play a definite part in the placebo effect. It is therefore possible that being enrolled in a formal research project in a university setting heightens participants' expectations of improvement, leading to an enhanced placebo effect and explaining, at least partially, the uniform response in the 3 groups. These non-specific effects would be principally attributable to the participants themselves and participant-provider interaction. The statistically significant recovery in the 3 groups in fear avoidance behaviour scores over time might be an indication of such positive participant-provider interaction.

Exercise adherence in the combined intervention group was 48.8%. This compares with previous estimates of adherence in home exercise programs for neck and low back pain, converging around 50%[42,43]. This may in fact reflect the highest level of adherence for these clinical populations. Considering the overall benefits and relatively low risk associated with exercise, clinicians may consider this therapy has an adjunct to SMT. However, several factors, such as frequent supervision and clarifications about exercises, are known to improve compliance and should consequently be considered in clinical trials involving exercise therapy[42].

### Comparison with other studies

Before the preventive phase of this study, all participants received a short course of treatment to relieve symptoms: SMT to the cervical and upper thoracic areas and myofascial soft tissue therapy when appropriate. All primary and secondary outcomes (pain, function and disability) demonstrated statistically significant improvement, except lateral flexion. The results of this non-randomised, symptomatic phase compare favourably to the existing literature on SMT for NCNP[44-48]. The symptomatic phase of our trial was unblinded and uncontrolled. It was, therefore, not possible to draw a specific conclusion regarding the causality of improvement, which might be attributed to one or a combination of treatment efficacies, the placebo effect, the natural history of the condition or the phenomenon of regression to the mean (spontaneous fluctuations in pain levels which tend, on average, to revert towards the mean). Furthermore, a more recent study[4] states that the evidence is often conflicting regarding the benefits of isolated manual therapy for NCNP.

Some trials have assessed the long-term efficacy and effectiveness of SMT for non-specific neck pain[44,49-51]. To our knowledge, this trial is the first to investigate the preventive influence of SMT with or without a home exercise program on NCNP. Given the present results and the innovative nature of the study, it is at this stage difficult to determine if the appropriate dosage of SMT was applied.

### Study strengths and limitations

The limitations of the present study include the absence of blinding of participants or treating chiropractors. This was inevitable due to the trial design (SMT for all participants in the symptomatic phase) and the impossibility of designing an adequate placebo for SMT[23,24]. Independent assessors, blinded to treatment allocation and clinical evolution, could have been involved in the trial. Physician global response assessed by a blinded evaluator should be considered in future studies.

A crucial aspect of any clinical trial is the equivalency of group sample size. In our RCT, randomisation was performed before the beginning of the trial's symptomatic phase, and an unequal number of drop-outs generated a smaller attention-control group than the experimental groups. It should also be noted that since the sample size of 35 subjects per group was not achieved, the pre-established statistical power was not reached. Because of the fluctuating nature of NCNP the patients included in this trial presented with low pain scores, at the time of enrolment. Such low levels of pain and related disabilities, may have limited the ability to detect statistically and clinically significant difference between the groups.

Another limitation is that during the preventive phase, participants in the attention-control group consulted only once every 2 months for a period of 20 to 30 minutes. This contrasts with the 2 experimental groups who consulted on a monthly basis for 10 to 15 minutes. The difference in protocol was deemed necessary to minimize drop-outs in the attention-control group. The actual dropout rate of the 3 groups was 11% for the SMT only group, 3% for the SMT combined with exercise group, and 10% for the attention-control group. Since the attention-control group did not incur a higher drop-out rate compared to the experimental groups, it can be concluded that this was an adequate strategy. However, the impact of such a strategy on clinical outcomes cannot be assessed independently of the present study.

Despite these limitations, this small-scale RCT demonstrates the feasibility of implementing controlled trials to investigate preventive strategies to improve prognosis or affect the natural history or recurrence of NCNP.

## Conclusions

The results of our study are important because there has been minimal previous research regarding the prevention of NCNP. Our results suggest that the mere fact of taking charge of and managing a patient for this condition might decrease the recurrence of pain episodes and, therefore, change the course of the disease. Considering the societal burden of NCNP,[1] the issue is worth investigating, both in terms of treatment efficacy and cost-effectiveness. Further research related to our hypothesis might be conducted in the form of a RCT.

Our results also indicate that there is no additional benefit for patients with NCNP to receive monthly preventive SMT or monthly preventive SMT with a home exercise program compared to meeting a chiropractor once every 2 months to discuss neck problems. In view of the rare but possible adverse reactions to cervical SMT, this tends to reject CPC when SMT is the main intervention. However, the premise of CPC stating that regular treatments, designed to preserve optimum health, will also minimize the recurrence of clinical problems, might hold true when intervention is geared towards reassurance, patient education, help with self-management and active care strategies. Further research in this domain has to be conducted.

A final implication of these results is the equivalence between the SMT and combined intervention groups. The actual, best evidence regarding treatment for NCNP is a combined approach involving manual therapy and exercise[4]. It is possible that the best strategy for prevention of NCNP might not be similar to the best strategy for treatment of this condition. Further research is warranted in this regard.

## Competing interests

The authors declare that they have no competing interests.

## Authors' contributions

MD contributed to trial design and protocol development, had overall responsibility for conduct of the trial, and contributed to recruiting patients, deciding the statistical analysis, interpreting the data and preparing the manuscript. CD contributed to trial design and protocol development, statistical analysis, data interpretation and manuscript preparation. JM was responsible for day-to-day management of the trial, follow-ups of the attention-control group, statistical analysis and manuscript preparation, and contributed to data interpretation. JDD participated in the development of the home exercise program used throughout the trial, was responsible of supervising the program execution by patients allocated to the SMT + exercise group and contributed to data interpretation and manuscript preparation. All authors had full access to all data (including statistical reports and tables) in the study and take full responsibility for data integrity and the accuracy of data analysis. MD is the guarantor. All authors read and approved the final manuscript.

## Pre-publication history

The pre-publication history for this paper can be accessed here:

http://www.biomedcentral.com/1471-2474/12/41/prepub
